# An adapted dorsal skinfold model used for 4D intravital followed by whole-mount imaging to reveal endothelial cell–pericyte association

**DOI:** 10.1038/s41598-021-99939-w

**Published:** 2021-10-14

**Authors:** Ann L. B. Seynhaeve, Timo L. M. ten Hagen

**Affiliations:** Laboratory Experimental Oncology, Department of Pathology, Erasmus MC, 3015CE Rotterdam, The Netherlands

**Keywords:** Collective cell migration, Cancer models

## Abstract

Endothelial cells and pericytes are highly dynamic vascular cells and several subtypes, based on their spatiotemporal dynamics or molecular expression, are believed to exist. The interaction between endothelial cells and pericytes is of importance in many aspects ranging from basic development to diseases like cancer. Identification of spatiotemporal dynamics is particularly interesting and methods to studies these are in demand. Here we describe the technical details of a method combining the benefits of high resolution intravital imaging and whole-mount histology. With intravital imaging using an adapted light weight dorsal skinfold chamber we identified blood flow patterns and spatiotemporal subtypes of endothelial cells and pericytes in a 4D (XYZ, spatial+T, time dimension) manner as representative examples for this model. Thereafter the tissue was extracted and stained as a whole-mount, by which the position and volumetric space of endothelial cells as well as pericytes were maintained, to identify molecular subtypes. Integration of the two imaging methods enabled 4D dissection of endothelial cell–pericyte association at the molecular level.

## Introduction

**Figure 1:**
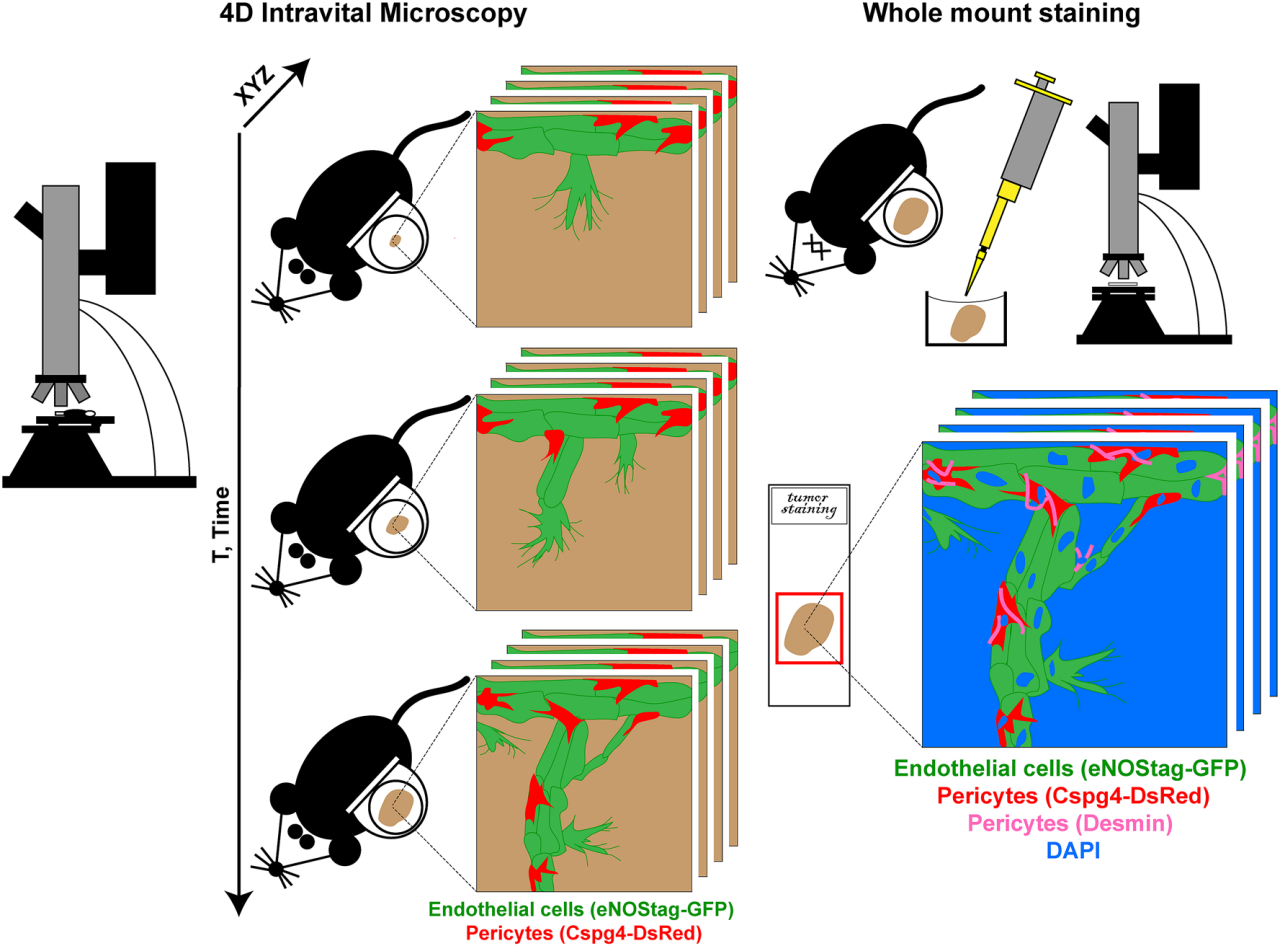
Schematic overview of the technique. High resolution 4D intravital microscopy is followed using whole-mount staining with the spatial positions still intact.

Angiogenesis, the formation of new blood vessels from existing ones, is a dynamic process in which endothelial cells and pericytes migrate, proliferate, form sprouts and make connections with other sprouts in order to form eventually a functional blood conducting network. Angiogenesis is tightly regulated and highly active during the embryonic stage until adulthood after which blood vessels remain predominantly quiescent with some exceptions like wound healing and during the menstrual cycle. Other angiogenic activations are found in pathological conditions such as inflammation, diabetic retinopathy and tumor growth. Research into the molecular and cellular processes of angiogenesis is not only of value for developmental biology but also for therapeutic purposes in these diseased settings^1–3^. Targeting the tumor-associated vasculature has been reported to be of great value in cancer therapy in which endothelial cells form the main focus as a clinical target. However, the role of pericytes in tumor development and therapy has gained interest over the last years^4,5^. In 2018 we introduced several transgenic animal models with fluorescent markers in two important cells of the vasculature (i.e. endothelial cells and pericytes), to investigate the dynamic association between these two cell types using intravital microscopy, making it possible to track the same region, vessel, cell and even cellular protrusions and organelles in a high resolution 4D manner^6^. When investigating a dynamic biological system, like angiogenesis, spatiotemporal data acquired from intravital imaging complements the results obtained using classical histology to investigate expression profiles and signaling pathways. However, classical histology requires sectioning of tissue and therefore compromises the position of individual cells. Sectioning can generate blind ending vessels which can be wrongly interpreted as, for example, endothelial tip cells or pericytes dissociated from a vessel. To really define and guarantee the position and identity of individual cells (e.g. endothelial tip cells, stalk cells, pericytes closely associated to the tip, pericytes in anastomosis) it is important to first identify cellular position and thereafter stain for additional cellular or molecular markers without tissue dissociation. In order to do so we first performed high resolution intravital imaging over time, identifying cells in positions of interest after which the tissue was stained as a whole-mount (Fig. 1). This was achieved using a transgenic mouse line in which endothelial cells as well as pericytes express a fluorescent marker, in combination with a tumor implanted in a modified light-weight dorsal skinfold chamber as an angiogenic model^6^. As we received since the publication many questions regarding technical details of the chamber model we focus in this report on the design of the dorsal skinfold chamber, the requirements of the equipment and the whole-mount staining procedure.

## Methods

### Ethics statement

Experiments involving mice and genetically modified organisms are performed according to all relevant governmental and institutional guidelines and regulations, in compliance with protocols approved by local animal ethics committees. Animal experiments, presented in this report, were approved by the Instantie voor Dierenwelzijn Erasmus MC and conducted with permissions granted by the Nederlandse Dierexperimentencommissie. The experiments were performed according to the European directive 2010/63/eu on the protection of animals used for scientific purposes and complies with the ARRIVE guidelines^7^.

### Animals and housing conditions

To allow visualization of endothelial cells and pericytes, transgenic animals with constitutive or inducible cell specific fluorescent markers are required. Tumor choice determines the genetic background of the animal and it is advised to cross the mouse line for several generations into the desired background to prevent variability. Hair is autofluorescent and, when using hirsute mice, hair at the back needs to be removed from the follicles by shaving and applying hear removal gel (VEET) preferably the day before surgery. The animals were housed in individual cages as, because of the gel, they smell differently and fighting can occur. Skin pigmentation does not present a problem for imaging. In this report we used offspring of a eNOStag-GFP^6^, expressing green fluorescence in endothelial cells and Cspg4-DsRed^8^ couple, or a Tamoxifen-induced Pdgfrb-CreERT2 and ROSA-mTomato couple^6^, expressing red fluorescence in pericytes, in a C57bl6 background and a B16BL6 melanoma or Lewis lung carcinoma implanted tumor. Mice should be 10 weeks or older and at least 25 g. After dorsal skinfold tumor implantation, the animals remained housed in individual cages, to avoid chewing on each other chamber, in a climate controlled environment set to 32 °C and above 50% humidity to keep the skin flap warm and moist.

### Design of the window chamber and adaptations to the microscope setup

**Figure 2:**
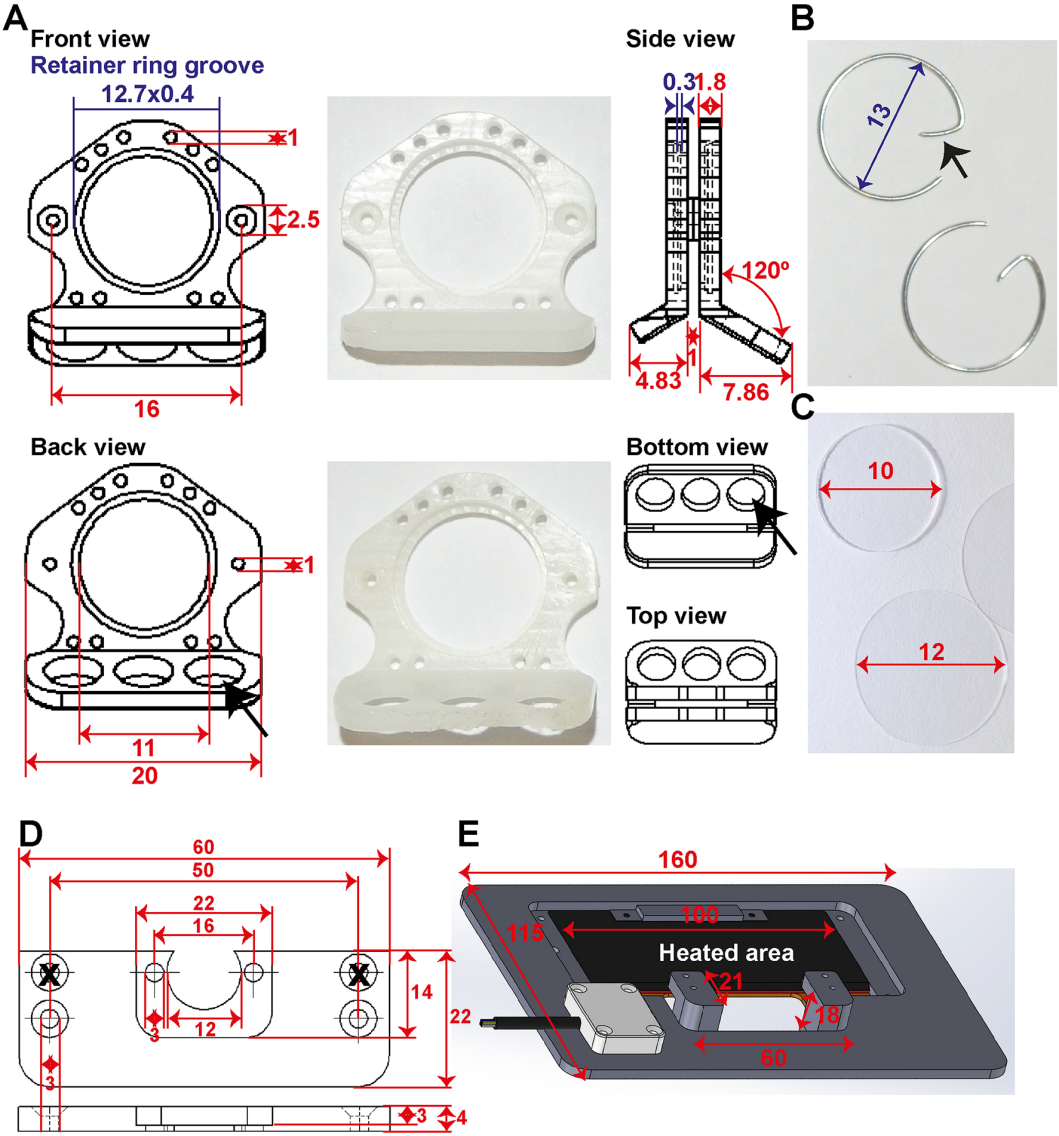
Design of the window chamber and adaptations to the microscope setup. Shown are the dimension in mm. (**A**) Shown here are the window frame, the window view area, sutures holes, and holes for the screws (in red). In blue are the dimensions for placement of the retainer rings. The groove is 0.4 mm thick and 0.3 mm deep inside both window frames. The bent hook of the front window is shorter compared to the back frame (see side view) for access of the objective lenses. The three big holes in the back frame (black arrow) are made for air circulation and simultaneous reducing the weight. (**B**) Retainer rings are made of flexible stainless 0.5 mm thick steel with a diameter of 13 mm. The hook (black arrowhead) serves for easy grip. Retainer rings without hooks can also be used when these interfere for instance with an objective lens. (**C**) A filler glass of 10 × 0.55 mm is used in the back to keep the skin close to the front glass. Both front and back window view area are closed with a standard 12 mm cover glass or in case of repeated evaluation with a 12 mm gridded coverslip. (**D**) Schematic overview of the chamber holder. (**E**) Schematic overview of the temperature-controlled chamber-to-stage platform.

We created window frames (Fig. 2A) from light weight polyether ether ketone (PEEK) and small retainer rings (Fig. 2B) to maximize the window view area. The weight of the entire chamber model presented here is, including glasses (Fig. 2C), retainer rings, screws and nuts 1.1 g. The retainer rings are home-made of flexible stain-less steel and the hook (Fig. 2B, arrow) functions as a grip for easy removal.

In this report we imaged using a SP5 upright multiphoton Leica microscope, although an inverted microscope, Zeiss LSM 510 Meta, is used by us as well ^9–12^. We adapted the current and previous microscope in such a way that these can be used also for histological sections or live cell imaging^13^. To fixate the chamber, while permitting the animal to breath freely, we constructed a two-unit platform to fit into the microscope stage. This platform consists of a chamber holder (Fig. 2D) to fixate the chamber and a temperature-controlled chamber-to-stage platform to place the animal under the microscope (Fig. 2E).

### Surgical procedure

**Figure 3:**
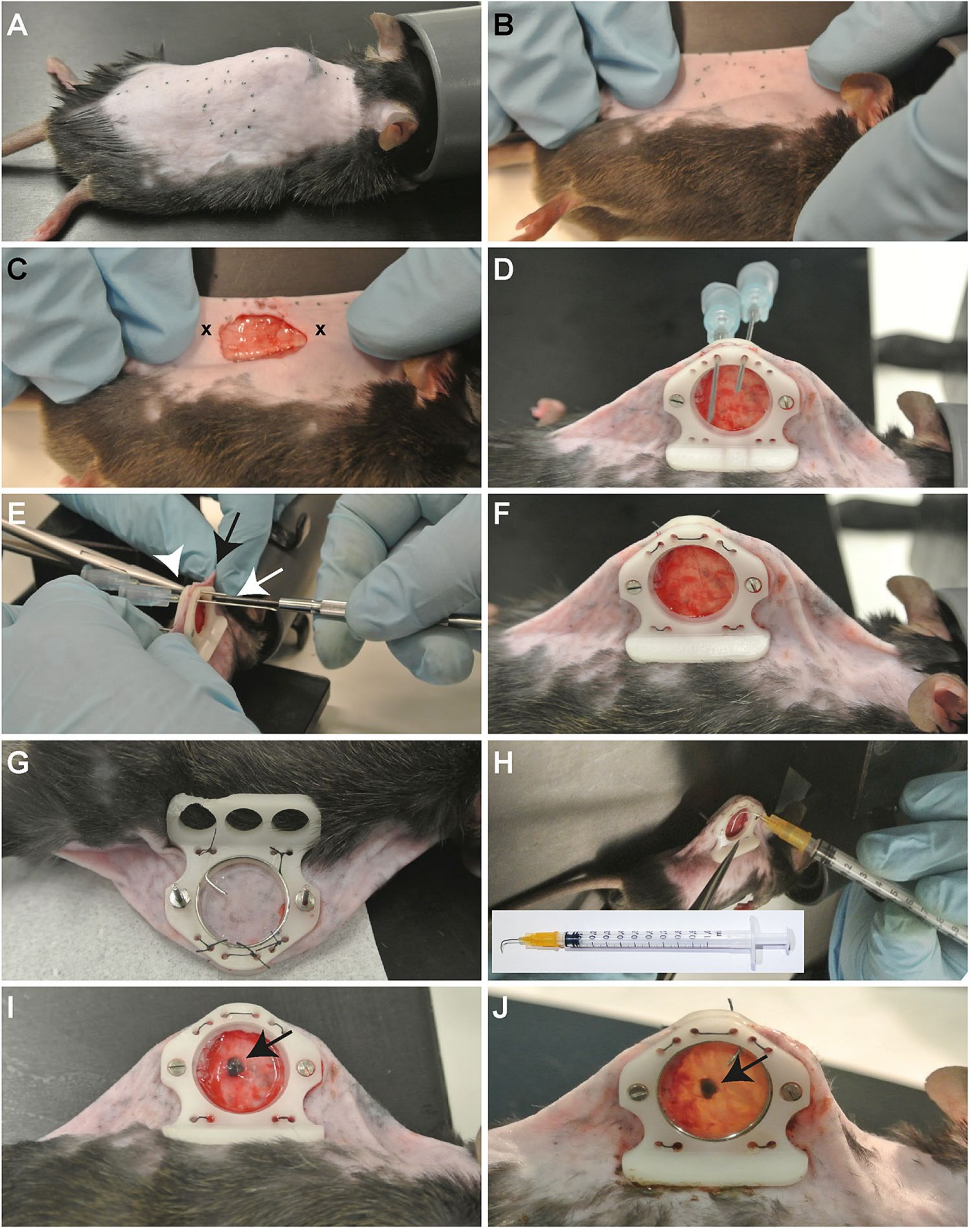
Surgical procedure. (**A**) The marked center line and window view area on the animal. (**B**) The skin folded using the central line as a folding point. (**C**) The exposed fascia after removal of skin and muscle layer inside the window view area. The cross (x) marks the spot to create the holes through the skin. (**D**) Front view of both window frames with screws and nuts (not yet tightened) and held in place with two 23G needles. (**E**) Tightening of the screws using a micro screwdriver (white arrow) and a needle holder (arrow head) to immobilize the nuts and holding of the skin (black arrow). (**F**) Replacement of the needles used to tighten the window frame with non-absorbable sutures and a knot on the backside and repeated for the other four pair of suture holes. (**G**) Back view of a 10 mm filler glass placed on the skin and closed with a 12 mm cover glass and secured with a retainer ring. (**H**) Creation of a pocket in the fascia using a 90° bent 25G needle for placement of the tumor piece. (**I**) The tumor piece (arrow) inserted in the pocket. (**J**) The end result after closing the chamber with a 12 mm blank coverslip or 12 mm gridded coverslip and secured with a retaining ring.

One hour before the surgical procedure the animal received 0.05–0.1 mg/kg buprenophine solution (Temgesic®; BD Pharmaceuticals) subcutaneously. The animal was anesthetized using isoflurane/O_2_ inhalation on a heated platform and ophthalmic ointment was applied. After disinfecting the skin with 70% (vol/vol) ethanol and surgical swaps the center line on the mouse spine and, 2 mm below this line in the middle of the animal, the circular window view area, using a window frame as a mold, was marked using a maker (Fig. 3A).

Using the center line as a folding point the animal was repositioned on the heated platform (Fig. 3B) and held in position using medical tape or a second pair of hands. Using a scalpel the marked window view area was cut and skin was carefully separated with the blade and holding the loosening skin with a micro forceps. Thereafter the separated skin was cut along the marked circular line with a micro spring scissor. The underlying fascia is now exposed and forms the transplantable area of the chamber (Fig. 3C). An operation microscope can be used for this procedure if needed. Thereafter the fold was repositioned upright and, using an earpuncher, two holes were punched (Fig. 3C, crosses) through the skin.

The screws, already inserted in the holes of the front window frame, were gently pushed through the holes in the skin. The back window frame was placed on the screws, and both frames were moved closer to the skin and nuts were placed loosely on the screws. Using a tissue forceps the skin was pulled 2–3 mm above the top of the frame and two 23G needles were pushed through the suture holes (Fig. 3D). The nuts were tighten with a micro screwdriver (Fig. 3E, white arrow) while preventing the nuts from spinning at the back by fixating the nuts with a clamping needle holder (Fig. 3E, arrowhead) and holding the skin to prevent it from turning around the screws (Fig. 3E, black arrow).

Thereafter the window was further fixated using sutures (non-absorbable, USP 6/0, metric 0.7; B. Braun Medical) by passing one end of the suture in the needle, pulling the needle out leaving the suture though the skin. When passing the other end of the suture though the other needle a surgical knot and an extra double wrap was made at the back. This was repeated for the four pairs of suture holes (Fig. 3F,G).

At the back (Fig. 3G), the fascia was moved forward by placing a 10 mm filler glass (Fig. 2C; diameter 10 mm, thickness 0.55 mm; Abrisa Technologies) in the window area, followed by a standard 12 mm cover glass (Fig. 2C; 12 mm, #1 thickness; Thermo Fisher Scientific) and secured with a home-made retainer ring (Fig. 2B).

The animal was repositioned to face the front area and sterile sodium chloride (0.9%; B.Braun) was dripped in the transplantable area. Using a bent 25G needle (Fig. 3H, insert), a small pocket in the fascia was made and, using a micro needle holder, a tumor fragment of 1 mm^3^ obtained from a donor tumor was inserted (Fig. 3H,I). Alternatively, tumor cell suspension can be injected into the fascia although this can create tumor spill in the transplantable area.

The chamber was closed with a 12 mm cover glasses and secured with a retainer rings (Fig. 3J). Alternatively, to realize reference points, a gridded round coverslip (12 mm; Electron Microscopy Science), which is originally designed for electron microscopy, was used to close the front view area. Within 8 h of surgery the animal received a second dose of buprenorphine. The animal needs to be checked regularly for chewed-off sutures, loose screws and nuts, broken cover glass and re-sutured, fasten and/or replaced when necessary (see trouble shooting).

### Intravital microscopy and imaging

**Figure 4:**
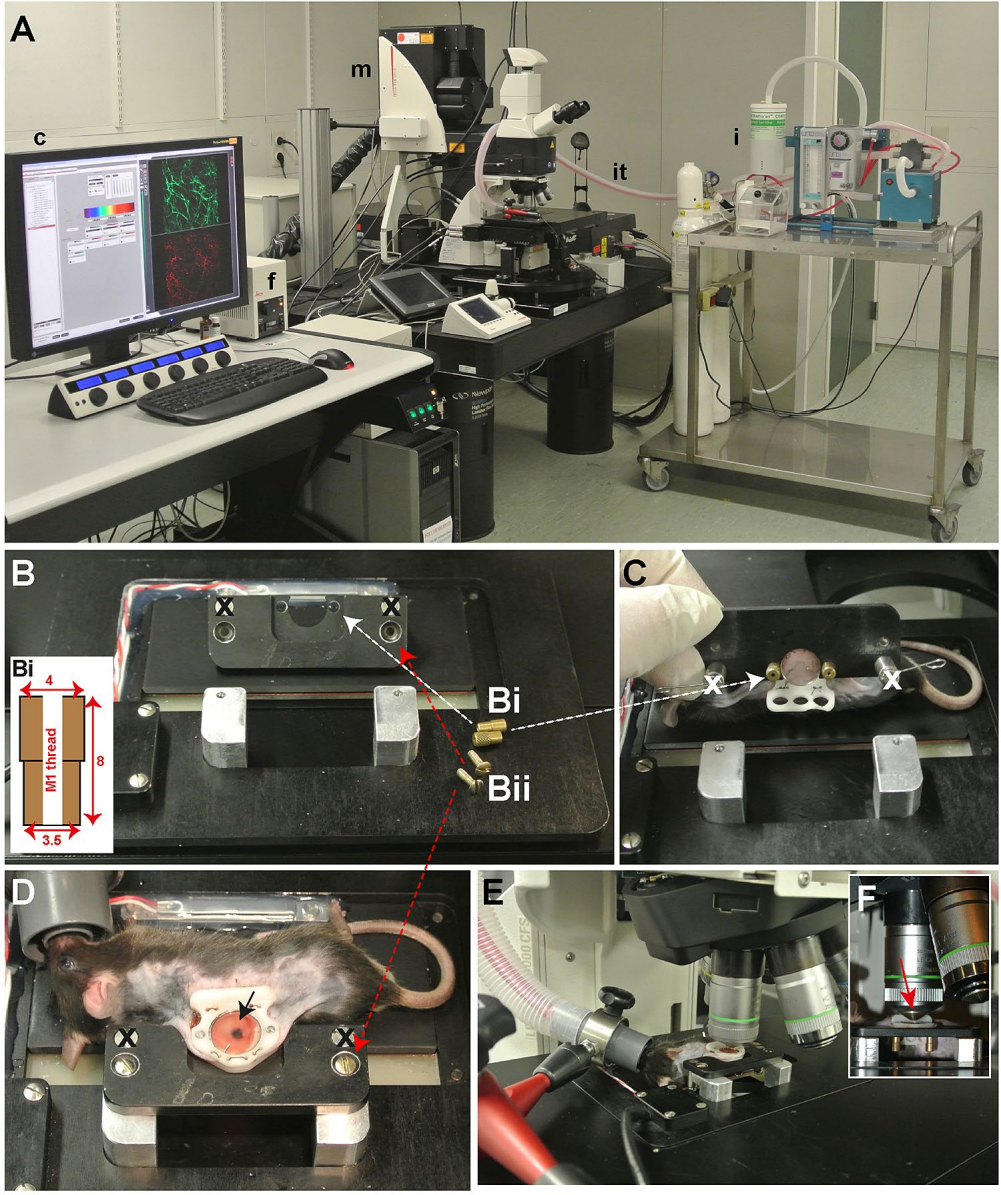
Intravital microscopy and imaging. (**A**) Equipment setup exist of a (c) computer with the appropriate imaging software (here LAS AF), (f) fluorescent light, (m) confocal microscope and an (i) isoflurane station with an (it) isoflurane tube connected to the microscope stage. (**B**) Chamber holder and chamber-to-stage platform secured with home-made knurled nuts (**Bi**, insert and white arrows) and standard 3 mm screws (**Bii** and red arrows) for securing the mouse chamber. (**C**) Back view of the chamber holder with the mouse chamber attached. (**D**) Top view of the mouse, fixated chamber with a B16BL6 tumor (black arrow) 11 days after implantation on the chamber holder and chamber-to-stage platform. (**E**) View of the animal on the microscope stage. (**F**) Side view of the tapered 20× objective (red arrow). The screws marked with the black or white crosses (x) are part of another model and are not required.

We imaged using a SP5 upright multiphoton Leica microscope (Fig. 4A). The most important requirement for the upright microscope is the distance between microscope stage and objective lenses; an animal has to fit in between. For a good quality image tissue stability is essential. The animal was anesthetized using isoflurane/O_2_ inhalation on the heated platform and ophthalmic ointment was applied. Using two homemade screws (Fig. 4Bi, white arrow) the chamber was immobilized via the screws of the window on the chamber holder (Fig. 4C). Thereafter the chamber was fixated using standard screws (Fig. 4Bii, red arrow) to the temperature-controlled chamber-to-stage platform (Fig. 4D) while the animal lies on its side on the heated part, which was thereafter placed in the microscope stage (Fig. 4E). For general visualization a standard 10× (NA 0.4) objective is sufficient and for detailed evaluation a tapered 20× (NA 0.5) objective lens is required (Fig. 4F, red arrow).

### Whole-mount staining to re-evaluate the tumor tissue

**Figure 5:**
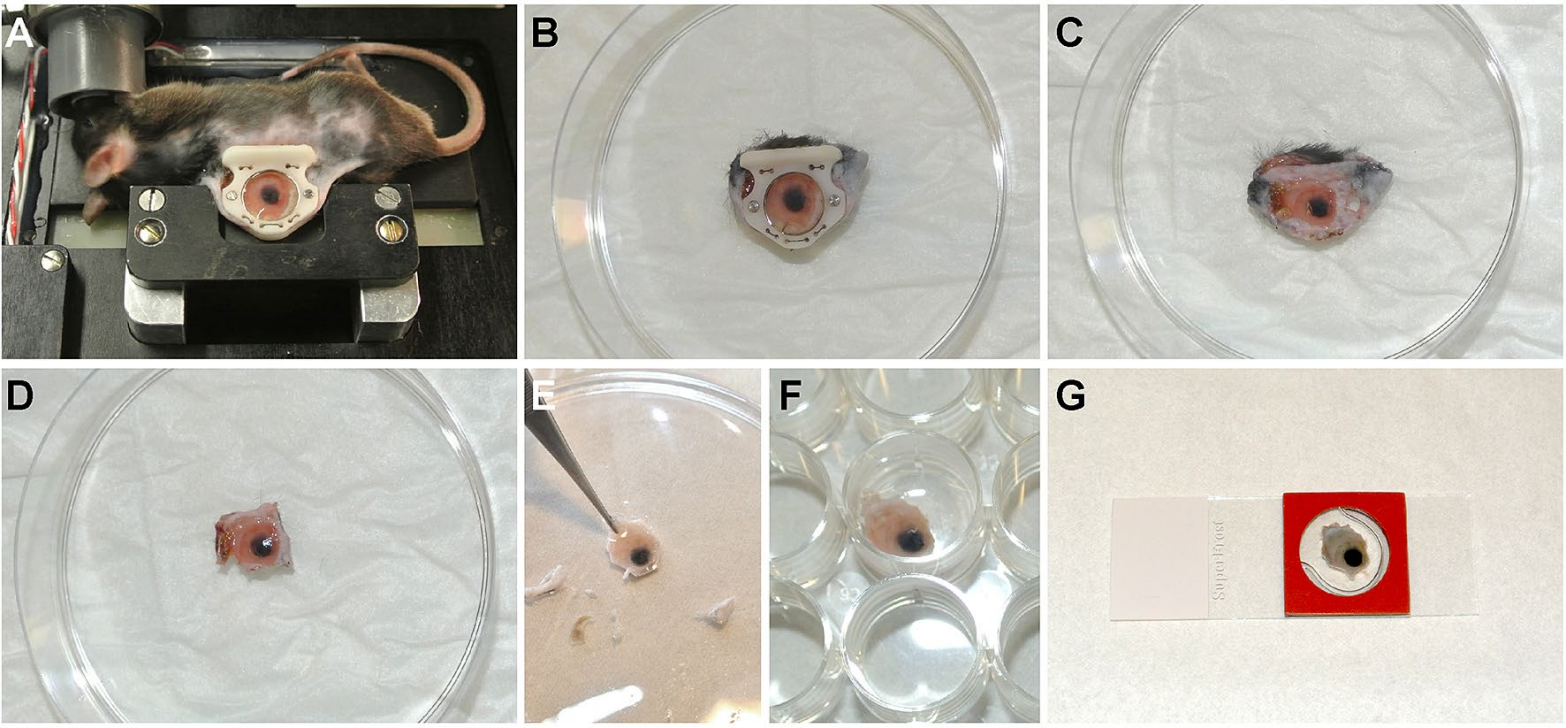
Whole-mount staining procedure. (**A**) Top view of the mouse, fixated chamber with a B16BL6 tumor 13 days after implantation on the chamber holder and chamber-to-stage platform. (**B**) After killing the animal the window is removed from the back of the animal. (**C**) Tissue without the window frames. (**D**) Excess skin removed leaving 5 mm tissue from the window view area intact. (**E**) Excess skin removed while leaving a piece of skin for easy handling (forceps position). (**F**) Staining of the tumor tissue in a 24 wells plate. (**G**) Mounting of tumor tissue using glycerol in an imaging chamber.

Classical immunohistology requires sectioning of the tissue compromising the position of individual cells. To investigate expression profiles and signaling pathways in locational defined subtypes it is therefore important to first identify cellular position and thereafter stain for additional cellular or molecular markers without tissue dissociation. This can be accomplished using intravital imaging followed with whole-mount staining of the entire window tissue. After intravital evaluation (Fig. 5A) and killing the animal, the window was removed (Fig. 5B) and the transplantable area including the tumor was dissected by removing the window frames and carefully lifting the front glass from the window view area (Fig. 5C). The tissue was fixed for 16 h to overnight in PFA (4% paraformaldehyde in PBS, pH 7.8; Sigma-Aldrich) at 4 °C. Paraformaldehyde was removed by washing the tissue with PBS for minimal 4 h at RT while shaking and refreshing PBS every 15 min. The excess skin was cut away so the transplantable area with the tumor fited unobstructed into the well of a 24 wells plate (Fig. 5D-F). Thereafter the tissue was permeabilized with PTT (0.3% Triton X-100, 0.1% Tween-20 in PBS; Sigma-Aldrich) for 48 h at 4 °C, blocked with PBT (4% BSA, 0.3% Triton X-100 in PBS; Sigma-Aldrich) for 48 h at 4 °C, stained with a first antibody diluted in PBT for 72 h at 4 °C, washed with PT (0.3% Triton X-100 in PBS) for 48 h at 4 °C and incubated with the corresponding 2nd antibody and DAPI (Sigma Aldrich) diluted in PBT for 72 h at 4 °C and washed with PT for 48 h at 4 °C. In the representative figure rabbit anti Desmin (Abcam) counterstained with an Alexa Fluor donkey anti goat 647 (ThermoFischer Scientific) was used. Other antibodies (Suppl. Table S1) and the commercially available kit for EdU staining (“Suppl. Method”) were also tested. The tissue was mounted with warm glycerol-gelatin solution (Sigma-Aldrich) sandwiched between two Grace Bio-Labs CoverWell imaging chambers of 20 × 1.3 mm (Sigma-Aldrich) and attach with superglue to a standard microscope slide (Fig. 5G). After solidification of the glycerol-gelatin solution the slide was kept at 4 °C for at least 24 h. We used the same SP5 Leica microscope to re-evaluate the whole-mount.

### Data analysis and statistics

Fiji (Wayne Rasband, NIH) was used for data analysis. To calculate velocity of individual cells images were taken at 0, 6, 12 and 24 h. Cells were selected at 0 h and the distance was tracked in the images taken at 6, 12 and 24 h. Velocity of vessel sprouts was determined by selecting sprouts and measuring the distance from the tip of the sprout to the first branching points in the sequential images. Velocity was calculated by dividing the recorded distance by time. To determine co-expression of pericyte markers, co-labeled cells were traced throughout the channels of the individual images of the Z-stack, using the nuclear staining to identify single cells and were counted manually. Results were evaluated for statistical significance with the Mann Whitney U test using IBM SPSS Statistics 21. P-values below 0.05 were considered statistically significant. Data was obtained from at least three individual experiments.

## Results

### Adaptations of the model and ethical consideration

**Figure 6:**
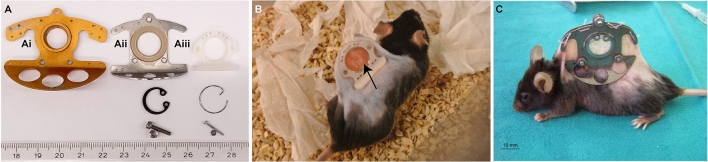
Differences in frame design. (**A**) Shown is the window frame developed by Jain et al. (**Ai**), our first adaptation (**Aii**) similar to the design of Dewhirst et al.^15^ and our current design (**Aiii**), including retainer ring, screw and nut. (**B**) Photograph of a conscious 25.1 g (excl. window) animal 2 days after implantation with a LLC tumor (arrow). (**C**) Photograph of an animal with a traditional window chamber taken from the publication from Regelin et al.^14^.

The classical dorsal skinfold model (Fig. 6Ai) was adapted to reduce the size (Fig. 6Aii), which is still used in a comparable format^14,15^. However, our current window model is even smaller and made of PEEK (Fig. 6Aiii). PEEK is resistant to most chemicals, inert, sturdy and autoclavable, does not provoke immune reactions and is 3.4-fold lighter than titanium. As a results of the smaller size of the frames, while retaining the same window view area, also the retainer ring, screws and nuts are smaller. Because of the reduced size and weight of the entire chamber animal welfare is increased improving the ethical consideration by the animal committees (Fig. 6B versus C). Flipping of the skin flap and animals treading on this flap, what happens when animals carrying heavy titanium windows for longer period, does not happen. Mice fitted with this window show full capacity of motion, climbing, and gain weight comparable to mice without a window chamber and infection of the window is rare. Skin on either side of the frames is not stretched to its limits and, although we use animals of at least 25 g for our experiments, mice of at least 17 g have also been used successfully in collaborating studies^16^. The animal may bite on the frames incidentally and frames are discarded when the edges are getting rough. However, the frames are easy to make and are inexpensive. Furthermore, if the hook of the retainer ring interferes with certain procedures, hook-less rings can be used. Window-carrying animals are MRI compatible as retainer rings are easy replaced by synthetic rings, while nuts and screws can be removed as also sutures can be used to fixate the frames^17^. Over the course of several days the tumor will grow in this area allowing high-resolution longitudinal evaluation.

### Requirements for good quality spatiotemporal images

**Figure 7:**
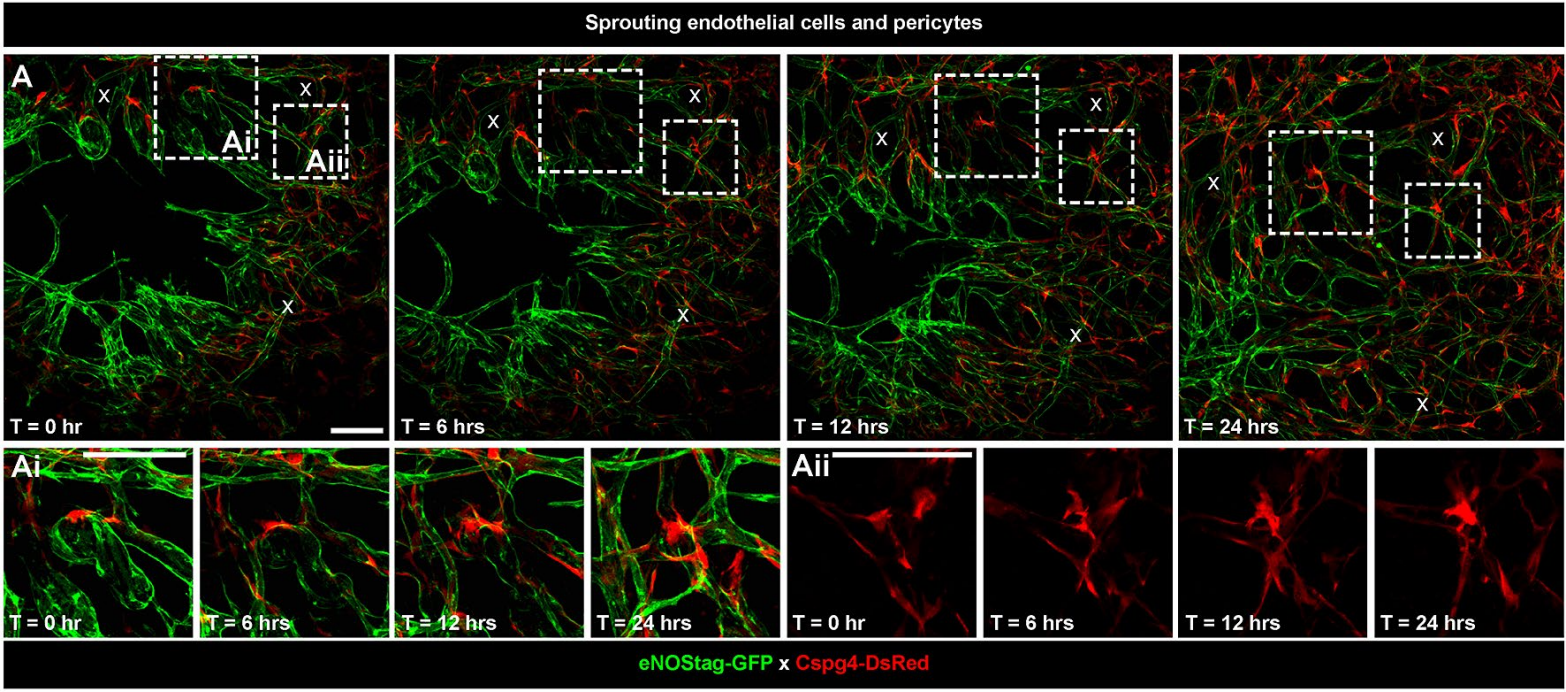
High resolution 4D intravital imaging of sprouting endothelial cells and pericytes. (**A**) Representative 70 µm subsequential maximal projections of endothelial cells (eNOStag-GFP in green) and pericytes (Cspg4-DsRed in red) in a B16BL6 melanoma tumor. (**Ai**, **Aii**) Zoom-in showing endothelial cell and pericyte spatial and temporal dynamics. x represents reference points in the vasculature. Scale bar represent 100 µm.

Spatiotemporal observations of cellular dynamics require imaging over multiple hours or even days. With several precautions like reduced isoflurane flow and subcutaneous hydration animals^18^ can be imaged continuously for up to 8 h. For multiple days evaluations, in which the animal was removed from the stage and re-evaluated later, reference points were required which can be done using the gridded coverslip in which the grid was made visible with reflected light (Suppl. Fig. S1). Using this grid makes it easier to find the correct positions back especially in fast growing tumors in which the tumor-associated vasculature can change drastically within a few hours (Fig. 7A). Also, reference points were presented in branching points of the vasculature and/or already quiescent established mature vessels (Fig. 7A, marked with x).

For image presentation and analysis a good signal-to-noise ratio, with qualitative high-resolution images, while simultaneously avoiding photobleaching, phototoxicity and saturation is a prerequisite. When using an upright microscope objective lenses need to have an unobstructed access to the window. The stage of tumor vessel development was checked using a 10× objective lens and fluorescent light and carry out further when vessel ingrowth was observed. When no vessels could be observed or the view was too blurry, this means that ingrowth of the vessels in the tumor had not yet started. In that case the animal was let to recover and re-evaluated the following day. A 10× objective lens was used for overall evaluation and a tapered 20× objective lens for detailed imaging. Setting the requirements for fluorescent imaging were not different from the requirements for standard immunofluorescent imaging. While avoiding over- or under exposure, the spectral range corresponding to the spectral profile of the fluorescent signals was applied, intensity parameters as pinhole, gain and offset was set and optimal signal-to-noise ratio by setting the image resolution, speed and average number of scans per focus point was determine. In general, a resolution of 512 × 512 or 1024 × 1024, a speed of 200 Hz and a line average of 4, gave good quality pictures with minimal bleaching. Also, when evaluating thick tissue, or to image an entire cell, a Z-stack needs to be taken. When using the gridded coverslip the grid was made visible using reflection of the 633 laser with low power (4%) on the glass (Suppl. Fig. S1). As this is just for reference the quality of the image can be low (i.e. format of 512 × 512, speed of 400 Hz and line average of 2). Besides intrinsic fluorescence also fluorescent makers were injected intravenously and long circulating fluorescent PEGylated nanoparticles^9,13^ were used as a blood marker.

### Hints for troubleshooting

A bleeding and/or air bubble occurs in the transplantable area during or after surgery: a vessel in the skin was cut and there was some air in the space between fascia and cover glass. The procedure can continue as blood and air bubbles are cleared away within 24 h.

#### Tumor is not growing

Tumor growth should become visible by eye 7–10 days after transplantation. The tumor does not grow when (1) a necrotic tumor fragment was transplanted, (2) the fascia was severely damaged or (3) environmental conditions were not correct. This means the end of the experiment and sacrifice of the animal. The tumor fragment needs to come from a viable part of the donor tumor, the fascia should not be damaged and the housing temperature of the mouse should be 32 °C and humidity above 50% to avoid cooling down and dehydration of the skin flap. Also fresh tumor material is recommended as success rate will drop significantly when transplanted tumor material comes directly from liquid nitrogen storage.

#### Post-surgery

(1) Fracture of the cover glass and/or (2) loose sutures: Mice are very flexible and these problems occurs when they are scratching and chewing on the glass, sutures and nuts. The mouse was anesthetized. (1) All glass fragments were carefully, not to damage the facie and tumor, removed and closed with a new glass. Bleeding can occur and should normally be cleared within 24 h. However, when fracture of the glass occurs during the imaging period, reference using the grid is not possible anymore. (2) The loose sutures were replaced and nuts tighten up. The animal should be given enrichment material to chew. Also, a cassette, made of light plastic, can be placed over the window.

#### Poor image quality

(1) The objective needs to be clean. (2) The cover glass of the window needs to be cleaned carefully using a cotton tip dipped in sterile distilled water. (3) Breeding movement can cause blurry images and therefore anesthesia needs to be monitored throughout the imaging period and the chamber should be firmly screwed on the chamber holder.

#### No or weak staining

The antibodies were too diluted, did not penetrate deep enough or were not competitive with PFA. Antibody concentration, permeabilization, incubation time and PFA compatibility need to be tested.

#### Intrinsic fluorescence is not visible anymore

The intrinsic fluorescence in the animals used here remains intact after PFA fixation. However if fluorescence is broken down this can be restored using antibodies against the fluorescent proteins.

### Intravital evaluation

Using intravital microscopy, dynamic cells of interest can be followed continuously for several hours or subsequentially, in which the animal is removed from the microscope stage and re-evaluated later. Using reference points with the gridded cover glass or stable positions in the vasculature we can re-evaluated the developing tumor ranging from a few minutes until the tumor fills the entire window view area. For fast growing tumors, like the B16BL6 and Lewis lung carcinoma, used in this manuscript, this takes approximately 72–96 h after first appearance of vessel ingrowth.

Here (Suppl. Video S1) we show a continuous 8 h time-lapse evaluation of several tip endothelial cells forming new vessels, branching off and re-connecting with an already established vessel.

Besides monitoring individual cells, the spatiotemporal dynamics of associated cells, like endothelial cells and pericytes, can be investigated in high resolution using intravital microscopy. Here, we show a representative subsequential time-lapse of endothelial cell and pericyte sprouting into an un-vascularized part of a tumor (Fig. 7A). The endothelial sprout is moving forward followed by pericytes. We have shown in the original paper^6^ that this distance is quite specific and the speed by which pericytes moves alongside endothelial cells is consistent indicating an angiogenic pericyte behavior. Shown also is the morphology of individual pericytes in the more established vascular region and this can be quite dynamic as well (Fig. 7Ai, Aii). As endothelial tip cell move forward to form a new endothelial tube, so do pericytes at identical speed (Suppl. Figure S2A). However, in contradiction to endothelial cells, pericyte hardily associate with other pericytes and migrate seemingly as independent cells (Suppl. Figure S2B) and, as we showed previously, are more associated with the endothelial cell counterpart^6^.

**Figure 8:**
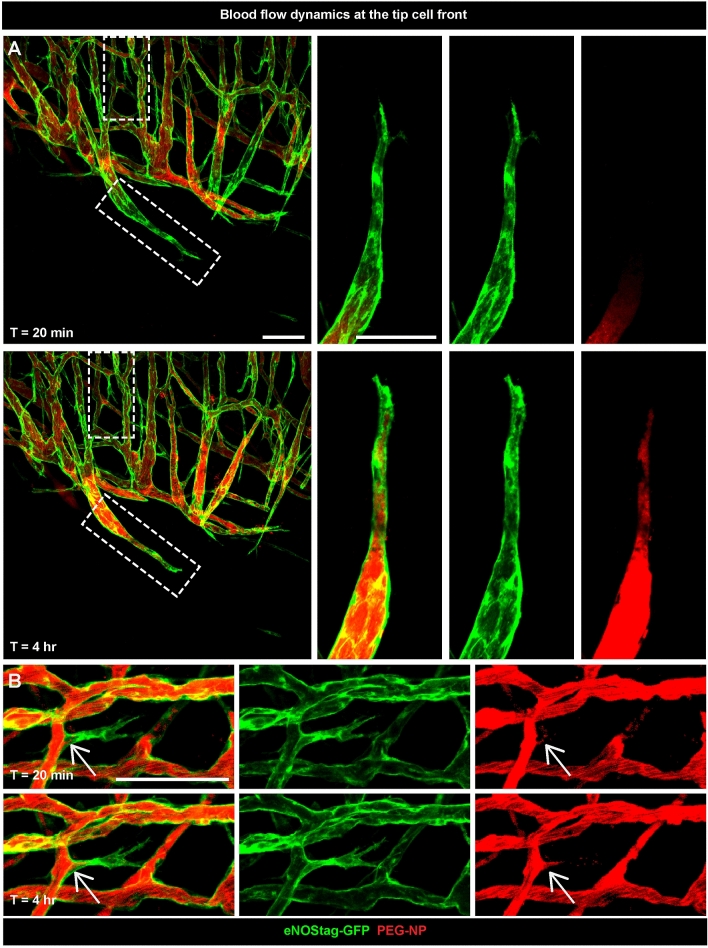
Blood flow dynamics at the tip cell front. Representative 70 µm subsequential maximal projections of endothelial cell (eNOStag-GFP in green) growth in a Lewis lung carcinoma and blood flow (Rhodamine PEGylated nanoparticles in red) 20 min and 4 h after injection of the particles. Scale bar represent 100 µm.

**Figure 9:**
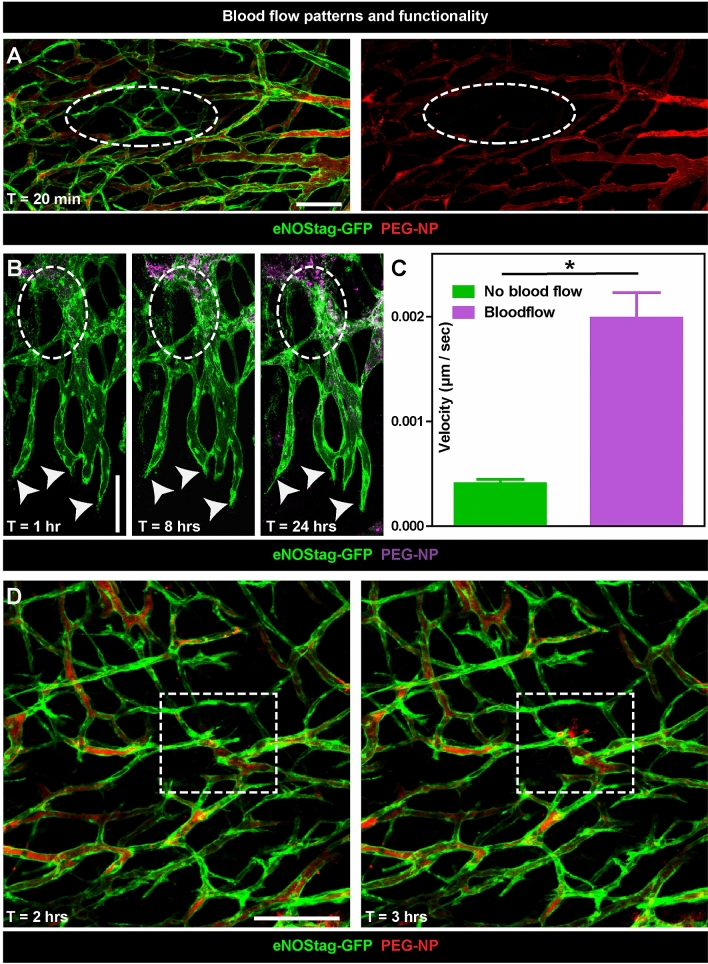
Blood flow patterns and functionality. (**A**) Representative 70 µm subsequential maximal projections of endothelial cells (eNOStag-GFP in green) in a Lewis lung carcinoma and blood flow (Rhodamine PEGylated nanoparticles in red) 20 min after injection of the particles. The dotted circle indicates a non-functional part of the tumor. (**B**) Representative 70 µm subsequential maximal projections of endothelial cells (eNOStag-GFP in green) in a B16BL6 melanoma tumor and blood flow (DiD PEGylated nanoparticles in purple) 1, 8 and 24 h after injection of the particles. The dotted circle indicates stalled blood flow followed by endothelial cell death identified by granulated cellular leftovers still fluorescent for GFP. Arrowheads indicate the lack of progression of endothelial tip cells. (**C**) Graph representing velocity of blood conducting versus non-blood conducting endothelial sprouts at the angiogenic front. Data represent average ± SEM of minimal five sprouts of three individual animals. *P < 0.05. (**D**) Representative still pictures of a sequential maximal projection of endothelial cells (eNOStag-GFP in green) in a Lewis lung carcinoma and blood flow (Rhodamine PEGylated nanoparticles in red) starting 2 h after injection. Dotted square focusses on vascular burst and extravasation of nanoparticles from the blood stream into the tumor interstitium. Scale bar represent 100 µm.

In addition to following cellular movement, we also monitored blood flow by injecting PEGylated long circulating nanoparticles (PEG-NP) labeled with a red fluorescent marker lissamine-rhodamine-phosphatidyl-ethanolamine (Rhodamine-PE) or far red fluorescent marker dioctadecyl tetramethylindotricarbocyanine perchlorate (DiD) ^9,13^. In these representative images we observed clearly the Rhodamine-PE red fluorescent marker in close proximity to the tip cell and continues to flow over time (Fig. 8A). Even in sprouts that are newly formed blood is already moving inwards (Fig. 8B). Secondly, blood flow represent functionality and also these effects can be investigated intravitally. The tumor-associated vasculature is “abnormal” in many aspects in comparison to normal vessels. The tumor-associated vasculature displays a lack of hierarchical branching organization in which the recognizable features of arterioles, capillaries and venules are lost. Vessels are tortuous and unevenly dilated. As a result, tumor blood flow is chaotic, can be stationary and even change direction as shown using bright field (Suppl. Video S2). This leads to hypoperfusion, hypoxia and acidosis in solid tumors. Also in areas with seemingly healthy lumenized vessels blood flow can be halted (Fig. 9A, circle). This is of particular interest in cancer therapy as these regions will not receive systemic administered agents, presenting a problem to obtain a more homogenous distribution which is necessary for successful therapy^9^. This is also represented here (Fig. 9B), as a part of the vasculature has no flow towards the tip cells area. When we re-evaluated this area 8 and 24 h later we observed that sprouting had halted (Fig. 9B, arrows) as velocity of these sprouts, measured between 0 and 8 h, was 4.8 lower compared to blood conducting endothelial sprouts (Fig. 9C), and parts of the vessels were destroyed (Fig. 9B, circle). This indicated a need for flow to promote or sustain angiogenesis. Also rare flow events, for instance vessel eruption and burst events, known to enhance delivery of nanoparticles into the tumor^19,20^, can be visualized using intravital microscopy (Fig. 9D, square and Suppl. Video S2).

### Re-evaluation

**Figure 10:**
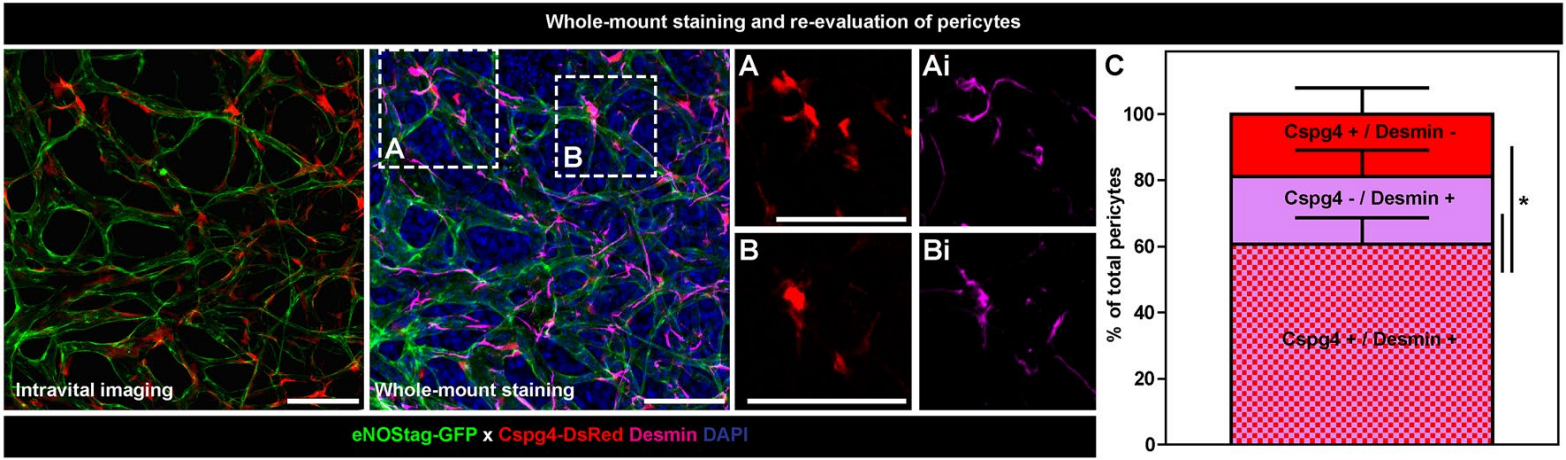
Whole-mount re-evaluation of pericytes. Representative 70 µm maximal projections of the intravital image and whole-mount staining. Endogenous fluorescence of GFP (eNOS, endothelial cells) and (**A**, **B**) DsRed (Cspg4, pericytes) is still present after PFA fixation and (**Ai**, **Bi**) pericytes are stained for Desmin (purple) and nucleus with DAPI (blue). Scale bar represent 100 µm. (**C**) Graph representing percentage pericytes expressing endogenous Cspg4 and stained Desmin. Data represent average ± SEM of minimal two position of at least three individual animals. *P < 0.05.

The molecular and biological mechanism and association between endothelial cells and pericytes is extensive^21,22^ and still not completely understood. Transgenic animals in combination with histology is a necessary tool to study these pathways. During angiogenesis endothelial cells are in general identify as endothelial tip cells, stalk cells and phalanx cells based on their spatiotemporal position and morphological appearance. Also pericytes subtypes express different cellular and molecular markers depending on their position (i.e. closely associated to the endothelial tip cell, involved in anastomosis, in branching points) and morphology, and staining with additional antibodies will help to identify cellular subgroups. In the previous report we stained the whole-mount for several pericyte markers^6^. The staining procedure of a whole-mount requires some optimalization and in the Supplemental information a table (Suppl. Table S1) is included for other antibodies. We also optimized the whole-mount staining for proliferating cells using EdU (“Suppl. Method” and Fig. S3). The representative figure shows the intravital image and the same position after whole-mount staining with desmin (Fig. 10). Cspg4 fluorescense is very condense in the cell and seen throughout the pericyte (Fig. 10A,B), whereas desmin is an intracellular filament protein and is seen as fibers thoughout the cell (Fig. 10Ai,Bi). We found that 61% ± 5 of the total number of pericytes are Cspg4 as well as Desmin positive (Fig. 10C).

## Discussion

Recently we published a paper introducing intravital evaluation of endothelial cells and pericytes using several transgenic animal models making it possible to track the spatiotemporal association between these two cells^6^. During angiogenesis, endothelial cells are, based on their spatiotemporal characterizes, subdivided into three cell types: tip, stalk and phalanx cells^22–25^. The endothelial tip cell, whether or not stimulated by flow, migrates forward into the tissue leading the new sprout, followed by proliferating stalk cells forming the vascular tube and the more quiescent phalanx cells in the already established vessels. In addition also pericytes can be subdivided according to their location in the vasculature^6^. In contradiction to the strong interaction between individual endothelial cells, pericytes associate with endothelial cells predominantly. Pericytes are, in positional space as well as in morphology, highly dependent on their endothelial partner^6^ and the signaling processes between both cells is extensive^26–29^ and still not fully understood. Investigating this cell type is challenging as there is no common pericyte marker and marker expression varies between species, tissues and conditions^30–32^, suggesting the existence of several subtypes. Here we describe in more detail the design of the window chamber we used, the adaptations made in the microscope setup and the whole-mount re-evaluation procedure. This is illustrated in the representative intravital and whole-mount images showing spatiotemporal evaluation of individual cells, blood flow patterns and cell–cell association and interaction. Furthermore, we show that whole-mount staining guarantees the correct spatial positions of endothelial cells and pericytes and enables identification of structures such as vascular spouts and tip cells. This is of particular interest as the dynamic nature of tip cell movement, tip and stalk cell competition, and the pericyte position with regard to endothelial tip cells, is still not clear^6,26,33,34^.

With the development of transgenic animals expressing cell specific markers, in combination with live cell tracers and better imaging equipment, intravital imaging has become an important tool to better understand these highly dynamic cellular processes. Besides intrinsic fluorescence also fluorescent makers were injected intravenously. For functionality of the vasculature Hoechst (Hoechst-33342; Invitrogen), a small 615 Da molecule, that diffuses rapidly into the tumor tissue and is taken up by the nucleus of surrounding cells^35^, can be injected. Also fluorescent labelled dextrans (Nanocs) in several sizes are available and dependable of their size extravasate in the tumor interstitium and are cleared from the bloodstream within a few hours^35^. Alternatively, if presence in the blood for more hours is desired long circulation fluorescent PEGylated nanoparticles^9,13^ can be used. Also fluorescently labeled chemotherapeutic drugs or therapeutic nanoparticles can be injected to observe treatment effects^9,10,13,36,37^. We have also used this method to locate engineered T cells to investigate T cell receptor gene therapy^38^. Also, to better investigate therapeutic effects intravital microscopy is now routinely used by our department. In addition, nanoparticles can be destroyed when using strong fixatives for tissue preparation, so also in this case using intravital microscopy is beneficial for a proper interpretation of the results. As we, next to the spatial position XYZ, also assess the time dimension T, intravital microscopy is in our laboratory often used to evaluate the therapeutic window, which is thereafter extrapolate to optimize tissue extraction for pharmacology and pharmacokinetics, significantly reducing the number of animals needed for these type of experiments.

## Conclusion

High resolution 4D intravital microscopy to evaluate cellular dynamics, like endothelial cell and pericyte association in angiogenesis and blood flow as presented here, is a very powerful tool. When integrated with whole-mount staining molecular and cellular processes can be re-evaluated without compromising spatial positions.

## Supplementary Information

Supplementary Information.

Supplementary Video S1.

Supplementary Video S2.
