# Supplementary material for: An adapted dorsal skinfold model used for 4D intravital followed by whole-mount imaging to reveal endothelial cell–pericyte association
Source: Sci Rep. 2021 Oct 14;11:20389. doi: 10.1038/s41598-021-99939-w (PMC8517006; doi:10.1038/s41598-021-99939-w)
Supplement: Supplementary file 1 — Supplementary Information. [file 41598_2021_99939_MOESM1_ESM.pdf]

An adapted dorsal skinfold model used for 4D intravital followed by whole-mount imaging to reveal endothelial cell – pericyte association.

Ann LB Seynhaeve, Timo LM ten Hagen

Supplemental information in order of appearance in the manuscript

Supplemental Table S1. Antibodies used for whole-mount staining

**Primary antibody**	**Company**	**Cat.no.**	**Usage**
Rabbit anti desmin	Abcam	ab15200	1:150
Guinea pig anti NG2	Donated by Prof. dr. Stallcup		1:800
Rabbit anti PDGFRb*	Donated by Prof. dr. Stallcup		1:250
Rat anti mouse PDGFRb*	eBioscience	14-1402	1:50
Mouse anti mouse SMA-Cy3*	Sigma-Aldrich	C6198	1:300
Rat anti mouse CD31	BD	553370	1:100
Rat anti mouse CD105	BD	550546	1:100
**Secondary antibody**	**Company**	**Cat.no.**	**Usage**
Donkey anti-rabbit IgG, Alexa Fluor 647	ThermoFischer Scientific	A31573	1:400
Goat anti-guinea pig IgG, Alexa Fluor 647	ThermoFischer Scientific	A21450	1:400
Donkey anti-rat IgG, Alexa Fluor 647	Jackson Immunoresearch	712-606-150	1:400
**Kits**	**Company**	**Cat.no.**	**Usage**
Click-it EdU Alexa Fluor 647 imaging kit	ThermoFischer Scientific	C10340	Below
**Other**	**Company**	**Cat.no.**	**Usage**
DAPI**	Sigma-Aldrich	D9564	1:1000

*PDGFRb and SMA antibodies also stain the capsule surrounding a tumor.

** Resuspend 10 mg DAPI in 2 ml distilled water and store at 4ºC

Supplemental Method. EdU labeling of tumor-associated cells

Dissolve 5 mg EdU in 2 mL NaCl for injections and inject i.v. 500 µg per animal as i.v. injection allows better penetration in the tumor tissue compared to i.p. injection. Kill the animal with cervical dislocation under anesthesia 4 hrs after injection. Use the procedure as indicated in the whole-mount procedure up to the permeabilization step with PTT. Remove PTT and wash with PT for 4 hrs. Remove PT, add Click-iT reaction cocktail according to the manufactures instructions and incubate for 72 h at 4°C. Remove reaction cocktail, wash with PT for 8 hrs and mount the tissue mentioned in the whole-mount procedure. Following this procedure GFP and DsRed signal are still visible in the tissue.

Supplemental Figure S1.

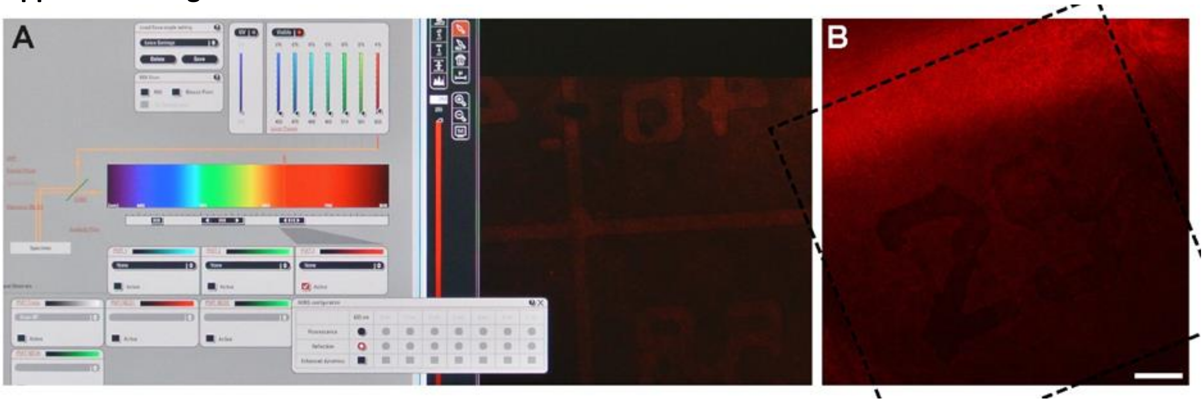

Reference point using a gridded cover glass. (A) LAF software view of the grid using reflection of the 633 laser on the glass. (B) Detail of the grid made with a 20x objective.

Supplemental Video S1: Sequential time-lapse of progressing endothelial tip cells in a B16BL6 melanoma tumor.

Supplemental Figure S2.

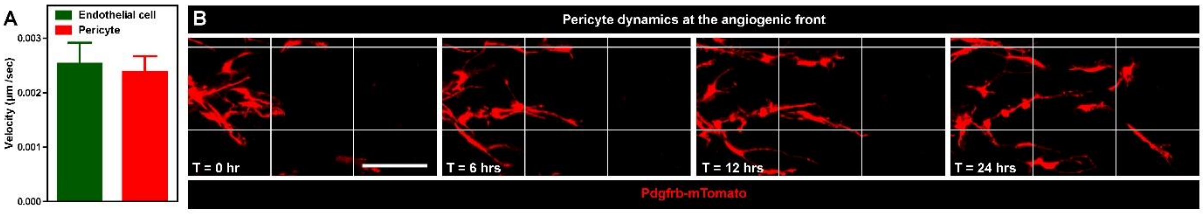

(A) Graph representing endothelial cell and pericyte velocity. Data represent average +/- SEM of minimal 5 cells of at least 3 individual animals. Data is not significant. (B) Representative high resolution 4D intravital imaging of pericytes at the angiogenic front. Shown are 70 µm subsequential maximal projections of pericytes (Pdgfrb-mTomato in red) in a B16BL6 melanoma tumor. Intersections are drawn to track travelled distance and calculate velocity. Scale bar represent 100 µm.

Supplemental Video S2: Sequential time-lapse of nanoparticle flow and extravasation in a Lewis lung carcinoma tumor.

Supplemental Figure S3.

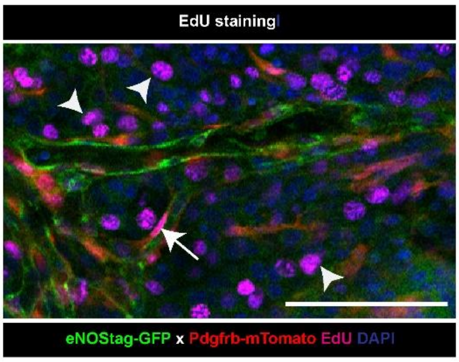

Representative single plane zoom-in image after whole-mount staining with EdU of a B16BL6 melanoma tumor. Endogenous fluorescence of GFP (eNOS, endothelial cells) and mTomato (Pdgfrb, pericytes) is still present after PFA fixation and proliferating cells are stained with EdU (purple) and nucleus with DAPI (blue). Arrow indicates a proliferating pericyte. Arrowhead indicates proliferating non-vascular cells, most likely tumor cells. Scale bar represent 100 µm.
